# Effect of Thermal Processing on Carotenoids and Folate Changes in Six Varieties of Sweet Potato (*Ipomoes Batata* L.)

**DOI:** 10.3390/foods8060215

**Published:** 2019-06-17

**Authors:** Zhijun Pan, Yiming Sun, Fangyuan Zhang, Xinbo Guo, Zhihua Liao

**Affiliations:** 1 School of Food Science and Engineering, South China University of Technology, Guangzhou 510640, China; 201664428579@mail.scut.edu.cn; 2 School of Life Sciences, Southwest University, Chongqing 400715, China; jwcsm@swu.edu.cn (Y.S.); fyzhang@swu.edu.cn (F.Z.)

**Keywords:** sweet potato, steaming, thermal process, carotenoids, folate

## Abstract

Carotenoids and folate are two mandatory supplying micronutrients for children or pregnant women. Inadequate intake of these two nutrients was relevant to a higher mortality of both children and pregnancies. This study is intended to investigate the thermal impact on the changes of carotenoids and folate in sweet potato roots (SPRs). Carotenoids were identified by high performance liquid chromatography (HPLC) while the folate was estimated using a microbial assay. An obvious decline was observed in total carotenoids after heating. Nevertheless, the content of provitamin compound β-carotene exhibited incredible stability during steaming and α-carotene multiplied in certain varieties, evidencing that SPRs could be an efficient way for addressing Vitamin A deficiency (VAD). As for the total folate contents, two varieties were found no significant loss after thermal process while the others showed a significant decrease. The results indicated that steaming process led to generally loss of both carotenoids and folate while the α-carotene and β-carotene were well preserved. The information provided by this study might help with enhancing the food quality in processing industry and the understanding in the nutrition changes during steaming.

## 1. Introduction

Carotenoids are one family of natural pigments and dietary micronutrients rich in natural foods. Dietary carotenoids can provide as vitamin A precursors to cleave vitamin A deficiency for human health. Vitamin A deficiency (VAD) is a serious worldwide public health issue that may lead to preventable blindness, impaired tissue function during the developmental period, and poor resistance to infection, affecting more than half of the countries in the world, especially low-income ones. Although a national vitamin A supplementation program has been around for years, the World Health Organization (WHO) estimated that there were still about 250 million preschool children suffering from VAD, particularly in Africa and South-East Asia [1]. Approximately, 250,000 to 500,000 vitamin A deficient children lost their sights every year and half of them died within 12 months [2]. Moreover, inadequate vitamin A intake during the last trimester for pregnant women was found had high correlation with night blindness and increasing the risk of maternal mortality. It was also suggested that VAD might contribute to affecting human immunodeficiency virus (HIV) through mother-child transmission [3]. Except for the function as provitamin A, a certain amount of studies revealed the positive relationship between high carotenoids intake and reducing the risk of developing degenerative chronic diseases, cardiovascular diseases as well as certain types of cancer including breast, cervical, ovarian, and colorectal. Additionally, the health benefits of skin protection, enhancing immunity, and promoting cognitive capacity were also found related to carotenoids intake [4]. 

As a great resource of carotenoids especially the provitamin A substance β-carotene with lower prices, sweet potatoe gained its important status in preforming vitamin A supplements for those developing countries whose diets lacked an animal food supplement [5]. In addition, considering of the high-production and high-energy-supply properties of sweet potatoes, cultivating biofortified sweet potatoes was also regarded as an effective mean for reducing poverty, ensuring household food security, and helping the country development. However, the overall consumption is low at around 1.1 kg/capita/year and mostly taken boiled or cooked without deep processing, figuring that there is still a large room for a further development in sweet potatoes processing industry [6].

Folate, also called vitamin B9, is another mandatory supplying micronutrient for human neural tube development. It is able to reduce the risk of affecting cardiovascular disease by decreasing the concentration of homocysteine [7]. In biochemistry, folate plays an essential role in transformation of certain amino acid and the synthesis of purines, which means folate is an indispensable part in DNA synthesis and cell proliferation [8]. However, limited studies focused on the changes of folate in sweet potato roots (SPRs) during thermal processing.

Several lines of studies have indicated the adverse correlation between steaming process and carotenoid content. A previous study showed that steaming process decreased α-carotene and β-carotene contents but enhanced (9Z)-β-carotene content in orange-flesh sweet potatoes [9]. Other studies have also demonstrated a negative effect of steaming on β-carotene content in orange-flesh sweet potatoes [10,11]. Literature about other substances in the carotenoid family (i.e., lycopene, lutein, etc.) in SPRs are infrequent. We assumed that these nutrients, which had many benefits for human health, were underestimated and required to be investigated in our study. As for the thermal effect on folate contents in SPRs, a few relevant articles were found. Sylvie Bureau et al. observed that folate content in frozen vegetables including green bean, broccoli, and cauliflower could be preserved by steaming [12]. Kariluoto et al. found that the thermal effect could cause a great loss of folate in rye [13]. However, there were limited researches demonstrated the thermal effect of steaming on those nutrients in SPRs, which made a difficulty in getting a better understanding of the nutrition quality in daily home cooking or food processing industry. In this study, six varieties of sweet potatoes roots (including pale, yellow, and orange colors) were selected to investigate the thermal impact on profiles of carotenoids and folate content in order to provide further information for nutritional changes of SPRs in the food processing industry.

## 2. Materials and Methods 

### 2.1. Samples Preparation

**Figure 1:**
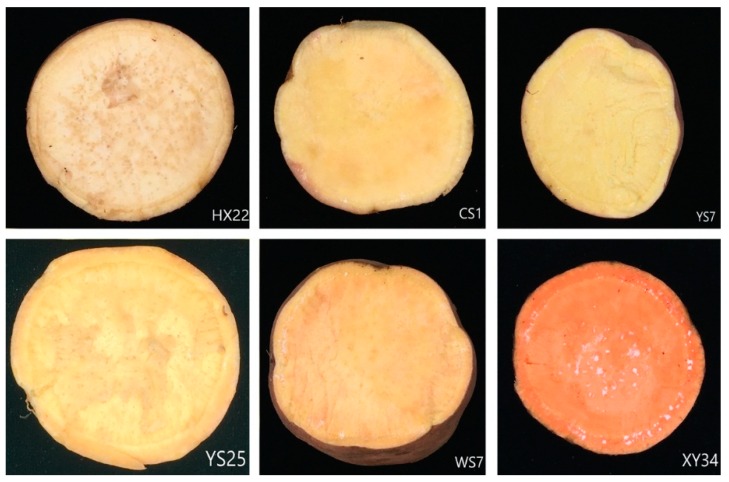
The six varieties of sweet potato roots.

The roots of sweet potatoes were supplied and identified by Dr. Fangyuan Zhang (Southwest University, Chongqing, China) including HX22 (pale color), XY34 (orange color), WS7 (yellow color), YS25 (yellow color), YS7 (yellow color), and CS1 (yellow color), as shown in Figure 1. After harvesting from the fields, the entire roots were washed carefully by hands with running water in order to wash away the dirt attached to the surface without peeling. Each root was cut in half and divided into two groups: steamed group and raw group. Different species of roots in steamed group were separated from each other by being wrapped in tinfoil. Then the steamed group was steamed on a steamer basket for 60 min at 100 °C. About 60 g of each variety from each group was cut into small pieces. The collected samples were treated with liquid nitrogen and grounded into flour then stored at −20 °C until analysis. 

### 2.2. Carotenoids Extraction and Analysis

The carotenoids in sweet potatoes was extracted by the method that has been reported previously, with slight modification [14]. For the extraction, a 1 g sample in triplicate was mixed well with a saponification agent. In the extraction process, the organic phases were collected and evaporated to dryness under nitrogen. The extracts were stored at −20 °C until analysis. Isomers of carotenoids were detected and evaluated by the high performance liquid chromatography (HPLC) method [14,15] with an ultraviolet detector at the wavelength of 450 nm and with the injection volume of 10 μL for each sample at the flow rate of 1.0 mL/min. The profile of carotenoids was completed by comparing the retention time and peak area between samples and standards. At last the result was also showed as μg/100 g of flesh weight sample (mean ± standard deviation (SD), *n* = 3).

### 2.3. Extraction and Determination of Folate Content

The extraction and determination of folate content in sweet potato root was carried out by the standard assay with modification that has been reported previously [16]. In brief, α-amylase, papain, and the chicken pancreas were used for extraction. DifcoTM Folate Assay Medium (Sparks, MD, USA) was used for cultivation. Enterococcus hire (ATCC^®^ 8043TM, American Type Culture Collection, Manassas, VA, USA) suspension was supposed to be prepared in advance for inoculation. To calculate the total content, the absorbance at the wavelength of 660 nm was recorded and performed by a standard curve made by folate pursed from Sigma, St. Louis, MO, USA. At last, the results were reported as μg folate per 100 g of fresh weight. (mean ± SD, *n* = 3)

### 2.4. Statistics Analysis

Statistical analysis was accomplished by OriginPro 2017 (OriginLab Corporation, Northampton, MA, USA). The significant test of difference was determined by Duncan’s multiple comparison with SPSS Statistic 22.0(SPSS Inc., Chicago, IL, USA). A p-value less than 0.05 was regarded as statistically significant.

## 3. Results

### 3.1. Thermal Effect on Carotenoids in Roots of Sweet Potatoes

The content of each subclass of carotenoids in each sample was calculated and presented in Table 1. The data were expressed as microgram per 100 gram in flesh weight (μg/100 g FW). Compared with the processed samples of the same variety, the raw samples performed a higher content of total carotenoids. As an orange type sweet potato, XY34 exhibited the highest content of carotenoids both in raw group and steamed group, followed by YS25, WS7, YS7, CS1, and HX22. Lutein was detected in all raw samples except CS1. However, it showed up in CS1 after steaming. The content of lutein significantly decreased by 100%, 64.76%, 65.24%, and 51.35% in HX22, XY34, WS7, and YS25, respectively and slightly increase by 6.8% in YS7. β-cryptoxanthin was only perceived in raw XY34, WS7, and YS25, as well as YS7, and was completely destroyed by thermal processes. As for the α-carotene, it was found in all the steamed samples except HX22 and showed a distinct increase by 171.21%, 100%, 212.84%, and 100% in XY34, WS7, YS25, and YS7, respectively, but slightly decreased in CS1 by 12.00%. β-carotene accounted for the most content of total carotenoids both in raw and steamed samples and amazingly showed the stability after thermal processing with slight decrease or increase in different varieties. (6R)-δ-carotene was simply found in two light-yellow varieties: CS1, YS7 while γ-carotene was merely observed in XY34 and reduced by 19.76%.

### 3.2. Thermal Effect on Folate Contents in Roots of Sweet Potatoes 

**Figure 2:**
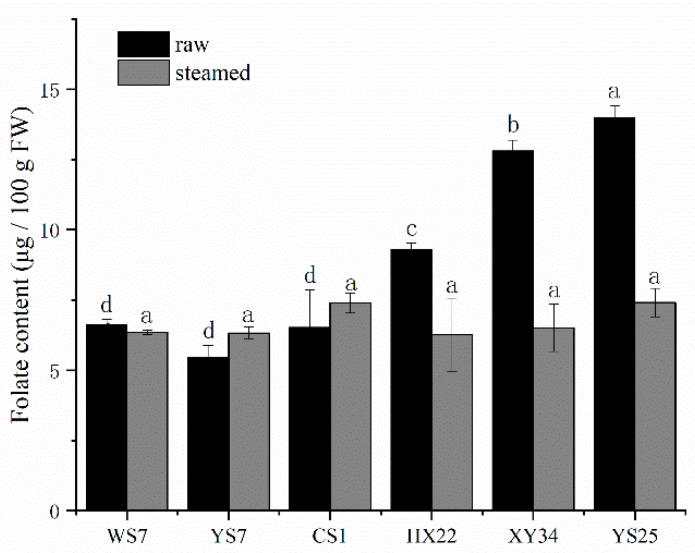
Effect of thermal processing on folate content changes in sweet potato roots (SPRs). Notes: Turkey tests were carried out in each row and bars with different letters differ significantly at *p* < 0.05.

The influence of steaming on total folate content (TFC) is represented in Figure 2 as microgram per 100 grams sample in flesh-weigh. TFC ranged from 5.44 to 13.99 μg/100 g FW in six varieties of SPRs. The raw YS25 sample had the highest content while the raw YS7 had the lowest. In addition, the content of the steamed samples showed no significant differences between varieties. With the exception of YS7 and CS1 (light-yellow type), all the samples showed a decline in the total folate contents after the thermal process. However, all the varieties still kept the TFC at the same significant level, around 5 to 7.5 μg/100 g FW, after steaming treatment. Moreover, the highest loss of TFC went to 47%, as compared with the raw samples of the same variety.

### 3.3. Correlation Analysis

**Figure 3:**
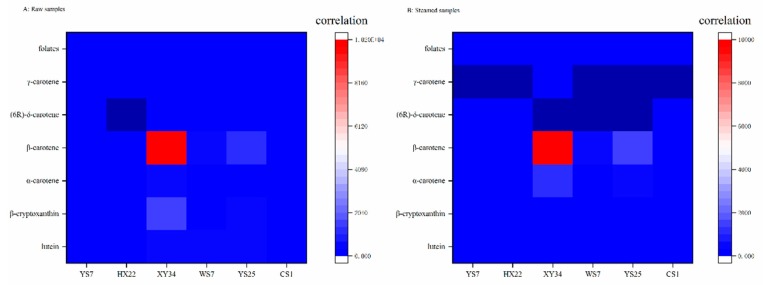
Correlation heatmap of carotenoids and folates in SPRs.

Table 2 and Figure 3 exhibited the correlation analysis results between the thermal process of steaming and the phytochemical compounds in SPRs. There showed no significant correlation between thermal process and the total content of folate as well as carotenoids. The total carotenoid content was found significantly relevant to β-cryptoxanthin (*p* < 0.05), α-carotene (*p* < 0.01), β-carotene (*p* < 0.01), and γ-carotene (*p* < 0.01) content while the TFC was relevant to lutein (*p* < 0.01) and β-cryptoxanthin (*p* < 0.01) content.

## 4. Discussion

### 4.1. Effect of Thermal Processing On Carotenoids

The current study supposed that by varying from varieties, each sample had its own unique composition of carotenoids [17]. Significant reduction of total content of carotenoids was detected in all selected varieties, irrespective of their flesh color and the phytochemical composition. The steaming process might have a negative effect on retaining the total content of carotenoids in sweet potatoes (Table 1). The incredible augment of α-carotene content in XY34 and YS25 should be noticed. Depending on the biosynthesis pathway reported by previous studies [4], we assumed that the lycopene ε-cyclase (LCYE) and lycopene β-cyclase (LCYB) were activated by heat treatment and then transformed the precursor substance of α-carotene such as all-trans-lycopene and δ-carotene into α-carotene. Actually, there was research on carotenoid accumulation in the postharvest of “Cara Cara” navel oranges, which were also considered to induce the gene expression of LCYE and LCYB [18]. More research would need to be done to illuminate the causes of this increase in XY34 and YS25. In contrast to the hypothesis that traditional cooking methods could enhance the bioactivities of β-carotene by breaking down the cell wall and releasing from the food matrix [10], β-carotene was found to increase or decrease in this study but showed an incredible stability during steaming. This finding may indicate that the same thermal process might make a different effect on different varieties and prove that SPR is an ideal crop for addressing VAD. β-cryptoxanthin, ε-carotene, and (6R)-δ-carotene were disrupted by degradation. The mechanism or the reason for this decline was still not clear and required a further investigation.

### 4.2. Effect of Thermal Processing on Total Folate Content

With the slight increase in YS7 and CS1 and decline in other varieties, the thermal effect on total folate content also varied from different varieties. According to the results presented in Figure 1, we speculated that the reason for the folate loss might be the vapor produced by the steaming process took away partial folate, given that it is a sort of water-soluble vitamin. Besides, thermal process might lead to degradation, accelerating oxidation actions on folate, which might be partially responsible for the decline. As for the gently augment of total folate content in YS7 and CS1, we assumed that there might be certain factors for preservation in those two varieties that could be activated by the steaming process. However, several lines of evidence indicated that steaming and a microwave process had no effect on the folate loss [19], which was in contrast to our findings. This indicates that further investigation is required in order to clarify the mechanism of the folate loss in SPRs.

## 5. Conclusions

Our research revealed the different influences on carotenoids and folate in various varieties of SPRs with a steaming process. The results showed that steaming tended to cause folate loss in certain varieties and carotenoids loss in all varieties. α-carotene showed an incredible increase after process. Additionally, β-carotene exhibited a stability with slight increase or decline in all varieties except CS1 and YS7 after the thermal process, evidencing that steaming is an ideal processing method for preserving β-carotene. Only two varieties—YS7 and CS1—did a good job of preserving folate during steaming. Our study stimulated the situation of daily cooking to process the samples in order to provide daily dietary guidance for consumers. The information provided by this study could also be applied to selecting better varieties for processing. Used as a vitamin A supplement, all varieties excluding YS7 and CS1 showed steaming stability of β-carotene might have a nutritional enrichment potential after processing and more suitable for processing before consumption. Further studies might provide a deeper understanding on the mechanisms behind these phenomena, as well as exploring key regulators and the nutritional fortification in food industry.

## Figures and Tables

**Table 1:** Changes of carotenoids profiles in sweet potato roots (SPRs) after thermal processing.

Carotenoids	HX22		XY34		WS7		YS25		YS7		CS1	
	Raw	Steamed	Raw	Steamed	Raw	Steamed	Raw	Steamed	Raw	Steamed	Raw	Steamed
lut	128.4 ± 7.4 ^g^	ND	492.4 ± 6.9 ^a^	173.5 ± 1.7 ^e^	377.3 ± 7.3 ^c^	131.1 ± 2.4 ^h^	450.1 ± 8.3 ^b^	218.9 ± 7.1 ^c^	139.7 ± 1.5 ^g^	149.2 ± 3.5 ^f^	ND	132.6 ± 5.7 ^h^
β-cry	ND	ND	1378 ± 60 ^a^	ND	246.4 ± 31.1 ^c^	ND	408.5 ± 14.3 ^b^	ND	127.1 ± 1.4 ^d^	ND	ND	ND
α-car	ND	ND	471.5 ± 16.4 ^b^	1279 ± 59 ^a^	ND	175.1 ± 9.4 ^d^	140.4 ± 0.2 ^e^	439.2 ± 14.2 ^c^	ND	72.13 ± 1.35 ^f^	71.18 ± 0.99 ^f^	62.64 ± 8.20 ^g^
β-car	133.9 ± 5.6 ^j^	138.2 ± 12.6 ^j^	10151 ± 217 ^a^	9964 ± 517 ^b^	451.7 ± 48.4 ^f^	462.6 ± 5.3 ^e^	1329 ± 216 ^d^	1383 ± 29 ^c^	193.1 ± 7.8 ^h^	155.8 ± 4.5 ^i^	239.2 ± 7.7 ^g^	136.2 ± 20.3 ^j^
δ-car	ND	ND	ND	ND	ND	ND	ND	ND	91.44 ± 3.03 ^a^	ND	89.68 ± 1.56 ^b^	ND
γ-car	ND	ND	158.8±2.5 ^a^	127.44 ± 1.6 ^b^	ND	ND	ND	ND	ND	ND	ND	ND
total	262.2 ± 13.2 ^k^	138.2 ± 12.6 ^l^	12625 ± 303 ^a^	11544 ± 579 ^b^	1075 ± 87 ^e^	768.8 ± 17.1 ^f^	2328 ± 239 ^c^	2025 ± 50 ^d^	551.3 ± 13.7 ^g^	377.3 ± 9.4 ^i^	400.1 ± 10.3 ^h^	331.4 ± 34.2 ^j^

Notes: Unit, μg/100 g FW. Turkey tests were carried out in each row and significant differences (*p* < 0.05) exist among those with different letters. ND means not detected. lut: lutein; β-cry: β-cryptoxanthin; α-car: α-carotene; β-car: β-carotene; δ-car: δ-carotene; γ-car: γ-carotene.

**Table 2:** Pearson correlation coefficient of carotenoids and folate content with thermal processing.

Coefficients	raw	steamed	lut	β-cry	α-car	β-car	δ-car	γ-car	TCC	folate
raw	1	−1.000 **	0.426	0.471	−0.297	0.006	0.447	0.049	0.040	0.458
steamed		1	−0.426	−0.471	0.297	−0.006	−0.447	−0.049	−0.040	−0.458
lut			1	0.772 **	0.181	0.455	−0.377	0.438	0.507	0.753 **
β-cry				1	0.142	0.618 *	−0.135	0.679 *	0.659 *	0.714 **
α-car					1	0.842 **	−0.231	0.760 **	0.822 **	0.067
β-car						1	−0.229	0.987 **	0.998 **	0.359
δ-car							1	−0.199	−0.234	−0.330
γ-car								1	0.986 **	0.354
TCC									1	0.401
folate										1

Notes: lut: lutein; β-cry: β-cryptoxanthin; α-car: α-carotene; β-car: β-carotene; δ-car: δ-carotene; γ-car: γ-carotene; TCC: total content of carotenoids. * and ** mean significant correlation at the 0.05 and 0.01 level, respectively (2-tailed).
